# Changes in responses of the amygdala and hippocampus during fear conditioning are associated with persecutory beliefs

**DOI:** 10.1038/s41598-024-57746-z

**Published:** 2024-04-08

**Authors:** Wisteria Deng, Lauri Tuominen, Rachel Sussman, Logan Leathem, Louis N. Vinke, Daphne J. Holt

**Affiliations:** 1 Department of Psychiatry, Massachusetts General Hospital, 149 13th, St. Charlestown, Boston, MA 02129 USA; 2 Department of Psychology, Yale University, New Haven, CT USA; 3 Department of Psychiatry, University of Ottawa, Ottawa, ON Canada; 4 Department of Psychiatry, Harvard Medical School, Boston, MA USA; 5 Athinoula A. Martinos Center for Biomedical Imaging, Massachusetts General Hospital, Boston, MA USA

**Keywords:** Psychology, Risk factors

## Abstract

The persecutory delusion is the most common symptom of psychosis, yet its underlying neurobiological mechanisms are poorly understood. Prior studies have suggested that abnormalities in medial temporal lobe-dependent associative learning may contribute to this symptom. In the current study, this hypothesis was tested in a non-clinical sample of young adults without histories of psychiatric treatment (n = 64), who underwent classical Pavlovian fear conditioning while fMRI data were collected. During the fear conditioning procedure, participants viewed images of faces which were paired (the CS+) or not paired (the CS−) with an aversive stimulus (a mild electrical shock). Fear conditioning-related neural responses were measured in two medial temporal lobe regions, the amygdala and hippocampus, and in other closely connected brain regions of the salience and default networks. The participants without persecutory beliefs (n = 43) showed greater responses to the CS− compared to the CS+ in the right amygdala and hippocampus, while the participants with persecutory beliefs (n = 21) failed to exhibit this response. These between-group differences were not accounted for by symptoms of depression, anxiety or a psychosis risk syndrome. However, the severity of subclinical psychotic symptoms overall was correlated with the level of this aberrant response in the amygdala (*p* = .013) and hippocampus (*p* = .033). Thus, these findings provide evidence for a disruption of medial temporal lobe-dependent associative learning in young people with subclinical psychotic symptoms, specifically persecutory thinking.

Prior neuroimaging studies of schizophrenia and psychosis risk syndromes have consistently detected anatomical and functional abnormalities of medial temporal lobe (MTL) areas such as the hippocampus and the amygdala^1–9^. Some studies have specifically linked MTL abnormalities in schizophrenia to the presence or severity of psychotic symptoms^10–15^, including persecutory delusions^12,16,17^. However, although findings of MTL abnormalities linked with psychosis have been well-replicated, it is unknown whether there are specific changes in cognitive or affective processes mediated by the MTL that contribute to the development of psychotic symptoms. It has been hypothesized that changes in the assignment of salience to incoming sensory stimuli by an MTL-striatal-midbrain circuit lead to abnormal perceptions and misinterpretations of those perceptions^18–20^, which then give rise to psychotic symptoms. The known central role of the MTL in associative learning and memory processes^21^ is consistent with this model, and prior studies have identified abnormalities in associative learning in individuals with schizophrenia^22–24^. However, it is unknown whether disruptions of associative learning processes mediated by the MTL are specifically linked to psychotic symptoms.

One form of associative learning that has been well-studied in animal models of psychiatric illness and in humans is Pavlovian aversive or threat conditioning, or what is commonly known as “fear” conditioning. This basic form of implicit learning is frequently measured in the laboratory using classical Pavlovian fear conditioning paradigms^25–28^. A recent meta-analysis that included 77 individuals with schizophrenia and 74 demographically-matched control subjects showed physiologic evidence for impaired fear conditioning in schizophrenia, particularly in those with active delusions^29^. Also, a prior study found some evidence for a role of the MTL in this abnormality, demonstrating hippocampal dysfunction during fear conditioning in schizophrenia^30^. However, the potentially confounding effect of treatment with antipsychotic medication in these prior studies limits their interpretability, particularly in light of evidence that D2 dopamine receptor blockade interferes with the acquisition or expression of conditioned fear responses in rodents^31^.

Therefore, to investigate the hypothesis that abnormalities in MTL-dependent associative learning are linked to psychotic symptoms, in the current study we employed a previously validated Pavlovian fear conditioning paradigm^32,33^ to measure MTL function during fear conditioning using fMRI. The participants of the study were young adults without a serious mental illness who endorsed varying levels of subclinical psychotic symptoms. Prior studies of fear conditioning conducted in healthy humans have shown that the human amygdala and hippocampus show conditioned fear responses^26,34,35^. Although the predicted pattern (based on rodent studies) of significantly higher responses to the CS+ compared to the CS− have been observed in the human amygdala^34,35^, more recent fMRI studies (including a meta-analysis) have also observed significantly larger responses to the CS− than to the CS+ in the amygdala and hippocampus of healthy human subjects^36,37^. This pattern of responses is consistent with the evidence that the amygdala and hippocampus are involved in both “threat” and “safety” -related learning^38–40^. Recent work has suggested that in a non-threatening context, safety signaling (i.e., CS− > CS+ responses) may dominate^39^, and a subset of safety-selective neurons in the amygdala may respond preferentially to the CS− during fear conditioning^40,41^. Given the prior evidence for abnormalities in fear and safety learning and memory processes in schizophrenia^29,30,42^, we hypothesized in the current study that such learning mediated by the MTL may be disrupted in individuals with subclinical psychotic symptoms.

Subclinical psychotic symptoms, often referred to as “psychotic experiences,” are common in the general population and are typically non-distressing and transient. Although the majority of individuals with psychotic experiences do not go on to develop frank psychosis, there is converging evidence, based on studies of epidemiologic and environmental risk factors for clinical psychosis^43^, as well as neuroimaging data^44–47^, for some degree of a continuum in the clinical and neurobiological expression of psychosis across different levels of severity in the general population, and for some biological mechanisms that are shared across psychotic experiences and clinical psychosis. One specific psychotic symptom that has been studied extensively across this continuum is persecutory beliefs, which can vary in severity from mild paranoia or persecutory thinking to full-blown persecutory delusions^48^. Since prior work has linked MTL dysfunction to the presence of persecutory beliefs or paranoia^16,44^, in the current study, we measured MTL responses during fear conditioning in non-help-seeking, young adults with persecutory beliefs. Based on this prior work, we predicted that we would observe evidence of abnormal MTL function during Pavlovian fear conditioning in those with persecutory beliefs, when compared to demographically-matched individuals without such beliefs.

## Methods

### Recruitment of participants

This study recruited and enrolled subjects via an ongoing study that focused on assessing various aspects of mental health in college students^49,50^ and the effectiveness of a behavioral intervention in this population^51–53^. In this parent study, we conducted in-person mental health screenings at three local universities over one or two days, in a high traffic area of the university. During the screening, study staff were available to consent participants and answer questions. Students who chose to participate in the overall study signed a consent form and completed a battery of self-report questionnaires.

For the current fMRI study, 72 non-help-seeking adults (65.6% female, age 18–25, mean age = 19.60) were identified as potentially eligible via these college campus screenings^44,54^ and then contacted and enrolled in the study. To recruit a mildly at-risk sample with a wide range of severity of psychotic experiences, as well as other types of psychopathology (e.g., depression), three groups of participants were enrolled: (1) those with an elevated score on the Peters Delusions Inventory (PDI,^55^; total PDI score > 7); (2) those with an elevated score on the Beck Depression Inventory (BDI,^56^; total BDI score > 5) but not an elevated PDI score (total PDI score < 8); and (3) those with low PDI and BDI scores (PDI < 8 and BDI < 6). These cut-offs of the PDI and BDI were chosen based on prior studies of these scales which indicated that these criteria identified approximately the top half of the distribution for depressive symptoms^57^, and subclinical psychosis^58^. Consistent with this, in our prior studies using these screening methods, these criteria typically identified approximately 20% (with elevated PDI scores) and 48% (with elevated BDI scores) of the distribution of the college students screened^49,50^. The goal of the study was to enroll young people with psychotic experiences and compare them to a control sample that also had a range of psychopathology, since psychotic experiences typically co-occur with other forms of psychopathology such as depression^59,60^. To test our specific hypothesis about persecutory beliefs, the enrolled cohort was divided into those who endorsed persecutory beliefs (one or both of the two persecutory items of the PDI) and those who did not.

Following quality control procedures, the data of 8 participants were excluded from the fMRI analyses due to excessive head motion during scanning (see criteria below). Thus, the data of 64 participants were included in the final analyses of this study (69% female, mean age = 19.7). All participants were proficient in English and had normal or corrected-to-normal vision based on Snellen test-based acuity^61^. At the time of enrollment, all subjects provided written informed consent. The study, including the experimental protocol, was approved by and conducted in accordance with the guidelines of the Partners Healthcare Institutional Review Board. The dataset analyzed is available from the authors by request.

### Clinical measures

Self-report measures of psychotic experiences including persecutory thinking, depression, and anxiety were collected, using the 21-item version of the PDI^55^, the BDI^56^, and the Spielberger State and Trait Anxiety Inventory State scale—state subscale (STAI-S^62^), respectively. These questionnaires were administered on the same day that the fear conditioning procedure (with simultaneous fMRI data collection) was administered. In addition, the Structured Interview for Psychosis-Risk Syndromes (SIPS) interview was administered to all participants, to determine whether participants met criteria for a psychosis risk syndrome^63^.

The 21-item PDI is a widely used self-report questionnaire designed to assess delusional ideation, including persecutory beliefs, and other unusual experiences in the general population. The PDI has been validated for use in clinical and non-clinical groups^64–67^. A total score is calculated by summing the number of endorsed items. We measured persecutory beliefs using the previously identified “persecutory factor” of the PDI^55,64,66^, which includes two items: “Do you ever feel as if you are being persecuted in some way?” (item #4) and “Do you ever feel as if there is a conspiracy against you?” (item #5). The cohort of 64 subjects with usable fMRI data was divided into those who endorsed at least one of these two items (n = 21, the “Pers” group) and those who did not endorse either of these items (n = 43, the “NoPers” group). Within the Pers group, 4 endorsed both items; 8 endorsed only item #4, and 9 endorsed only item #5.

The BDI is a well-established 21-item self-report measure of depressive symptom severity over the past two weeks. Participants rate the degree to which they have experienced each symptom on a four-point scale from 0 to 3. The STAI-S contains 20 questions that measure the severity of anxiety symptoms that individuals are experiencing in the present on a Likert scale from 1 to 4.

The Structured Interview for Psychosis-Risk Syndromes (SIPS) and the Scale of Psychosis-Risk Symptoms (SOPS,^63^), administered by trained post-baccalaureate interviewers with direct supervision by a licensed psychiatrist, were used to rate the severity of symptoms of schizophrenia experienced by the participants. The SOPS consists of 19 items in 4 symptom domains: positive, negative, general, and disorganized. The five positive symptom items are: unusual thought content, suspiciousness, grandiosity, perceptual abnormalities, and disorganized communication, each rated on a score of 0 (none to minimal) to 6 (present and psychotic in intensity), with ratings of 3 to 5 representing attenuated psychosis. In this sample, 11 of the Pers group and 10 of the NoPers group met Attenuated Positive Symptom Syndrome (APSS) criteria based on the SIPS/SOPS. The Pers and NoPers groups showed no differences in gender frequencies and mean age (Table 1). There were expected differences between the two groups in levels of symptoms of depression and overall psychotic experiences, with significantly higher levels (all *p*s < 0.05) in the Pers group.

**Table 1:** Participant characteristics.

	NoPers (n = 43)	Pers (n = 21)	*p* value
Gender (% female)	62.8	71.4	0.582
Age (years)	19.49	19.81	0.319
Depression	5.32	11.17	**0.002**
State anxiety	33.12	37.22	0.102
Psychotic experiences	2.88	6.26	** < 0.001**
% attenuated positive symptom syndrome	23.3	52.4	**0.026**

Mean values for the group without persecutory beliefs (NoPers) and the group with persecutory beliefs (Pers) are listed for the primary symptom measures assessed in this study. Depression was measured with the Beck Depression Inventory; state anxiety was measured with the State-Trait Anxiety Inventory; psychotic experiences (including delusional beliefs and some unusual perceptions) were measured with the Peters et al. Delusions Inventory; and the presence of the Attenuated Positive Symptom Syndrome (APSS) was measured using the Structured Interview for Psychosis-Risk Syndromes. P values for independent t-tests comparing the means of the two groups are listed. Significant p values are bolded. As expected, the Pers group had higher mean levels of depression and psychotic experiences and included a larger number of participants who met criteria for APSS than the NoPers group.

### Fear conditioning paradigm

All subjects underwent a Pavlovian fear conditioning procedure while fMRI data and skin conductance responses (SCRs) were collected simultaneously. For each subject, one face pair of two possible face pairs was selected (counterbalanced across subjects). One face of the face pair was assigned to be the conditioned stimulus (CS+) and the other was assigned to be the neutral (CS−) stimulus. The selection of face pairs and assignment of the CS+ and CS− to the two faces of each pair were pseudo-randomized across the subjects.

**Figure 1:**
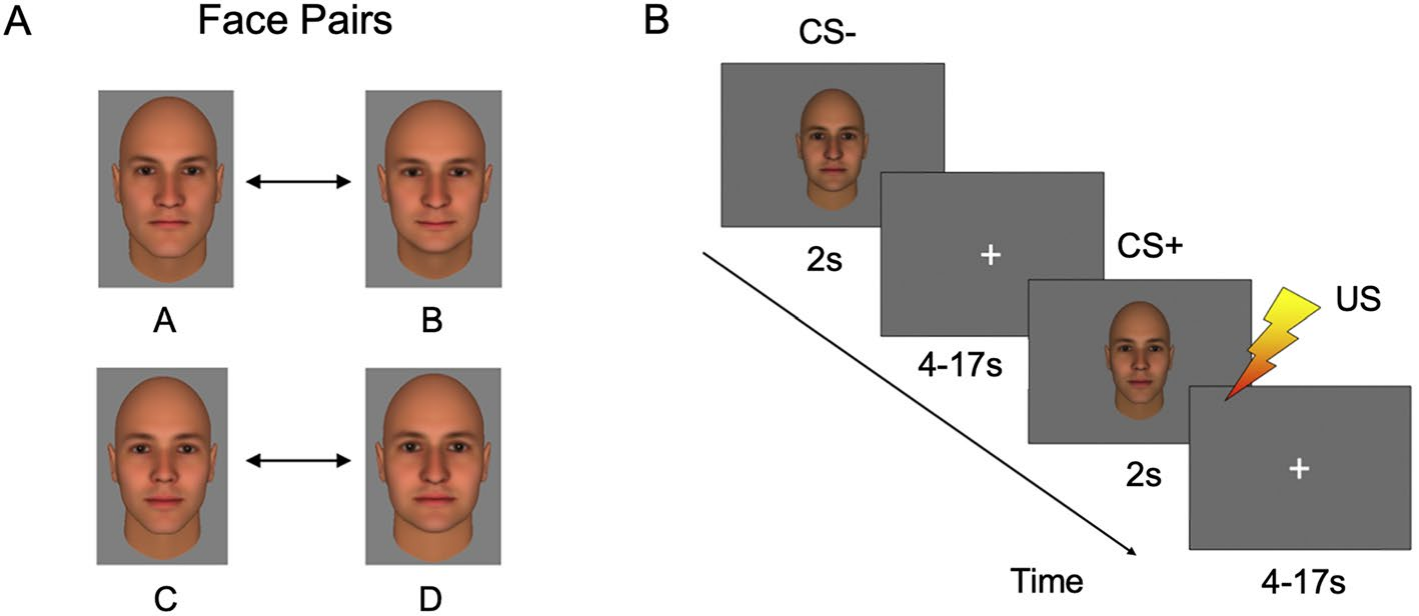
Schematic diagram of the fear conditioning paradigm. (**A**) For each subject, one of two face-pairs was selected (pseudorandomly, counterbalanced across subjects). One face per pair was assigned to be the conditioned stimulus (CS+) and the other was assigned to be the neutral (CS−) stimulus. (**B**) During the fear conditioning paradigm, 13 CS+ trials and 13 CS− trials, each 2 s long, were presented in a pseudorandom order. The inter-trial intervals (ITI) were between 4 and 17 s long. Following 8 of the 13 CS+ trials, the unconditioned stimulus (US), a 500 ms electrical shock, was delivered to the shin of the left leg.

During the fear conditioning paradigm, 13 CS+ trials and 13 CS− trials, each 2 s long, were presented in a pseudorandom order. The inter-trial intervals (ITI) were between 4 and 17 s in duration. The CS+ was immediately followed by a 500 ms long electrical shock (the unconditioned stimulus; US) that was administered to the shin of the left leg following 8 of the 13 CS+ trials. The intensity of the US ranged from 1.1 to 4 mA and was set individually to a level that was “highly annoying but not painful” to the participants prior to the procedure, as in previous studies^33,42,68^. To ensure that the participants were attending to the stimuli, a button-press task was performed during the fear conditioning procedure. During the procedure, 30% of the CS+ and CS− face stimuli appeared to briefly nod (for 500 ms) during the presentation. The participants were asked to press a button whenever they saw a nod. There were no significant differences between the Pers and NoPers groups in rates of detection of the nods (*p* = 0.86). See Fig. 1 for a schematic diagram of the paradigm and example stimuli. The fear conditioning phase was then followed by a fear generalization phase (findings for the fear generalization phase of this study will be reported separately).

### Skin conductance measurements

Skin conductance responses were measured using two MRI-compatible electrodes placed on the palm of each subject’s left hand at the beginning of the scan session. These data were recorded using a BIOPAC MP150 acquisition system (BIOPAC Systems, Inc, Goleta, CA) and an EDA100C MRI amplifier. Skin conductance was collected at a gain of 5 µS/V. During acquisition, data were low pass filtered at 1 Hz and digitized at 200 Hz.

### MRI data acquisition

All MRI data were collected on a 3T Siemens Prisma scanner using a 64-channel head coil (Erlangen, Germany) at the Athinoula A. Martinos Center for Biomedical Imaging. T2*-weighted echo-planar images were collected during the fear conditioning and generalization paradigm (2 mm isotropic, matrix = 64 × 64, 45 slices, TR = 2000 ms, TE = 30 ms, flip angle = 90°). In addition, a T1-weighted 3D scan was collected using an MPRAGE sequence (spatial resolution 1 mm isotropic, matrix = 256 × 256, 176 slices, TR = 2530 ms, TE = 1.64, 3.5, 5.36, and 7.22 ms, flip angle = 7°).

### Data analysis

#### Skin conductance analysis

Skin conductance data were analyzed using the ledalab V3.4.9 toolbox running on MATLAB R2016b (Mathworks; MA, USA). Data were downsampled into 10 Hz and manually cleaned for artifacts. A decomposition of the data into continuous signals of phasic and tonic activity was performed. A minimum amplitude threshold of 0.01 S, and maximum phasic values within response windows of 1–4 s after the events were extracted. To test for a fear conditioning response, the mean SCRs to the CS+ and CS− stimuli were compared using paired t-tests.

#### MRI data analyses

##### Preprocessing

Functional MRI data were preprocessed using the standard FSFAST processing pipeline in FreeSurfer version 6.0 (http://surfer.nmr.mgh.harvard.edu). Briefly, functional images were corrected for motion and slice timing and then spatially transformed into a common space (i.e., fsaverage on the cortical surface and MNI152 in the subcortical volume) and then spatially smoothed using a 3D Gaussian kernel (5 mm FWHM). A canonical hemodynamic response function was fitted to the CS+ and CS− events in each vertex and voxel. Temporal drift was accounted for by first- and second-degree polynomials in the first-level model. Motion parameters and timepoints with excess motion were used as regressors in the first level model.

##### Region-of-interest (ROI) analysis

The two primary ROIs, the amygdala and hippocampus, were defined based on anatomical criteria using each subject’s T1 anatomical scan, using the FreeSurfer segmentation algorithm^69^. In addition, six secondary ROIs were used in exploratory analyses of additional brain networks outside of the MTL known to play a role in fear conditioning. Five of these ROIs were defined using maps of fMRI responses to the CS+ v. CS− contrast generated using fMRI data collected in healthy control subjects in an independent fear conditioning study^33^. Three ROIs of the salience network (i.e., the anterior insula, dorsomedial prefrontal cortex (dmPFC), and thalamus) and two of the default mode network (i.e., precuneus and angular gyrus) were defined in this independent dataset. One additional ROI, the caudate nucleus, a key node of the salience network, was anatomically defined in each subject using the FreeSurfer automated segmentation procedure.

The ROIs defined in the previously collected fear conditioning data were constructed by identifying the border of each significant cluster of activation in that ROI (as identified by the FreeSurfer parcellation), at a threshold of *p* = 0.001, corrected for multiple comparisons using Monte-Carlo simulations with a voxel-wise *p* value threshold of 0.001, in the group template (fsaverage) space. Each of these template labels was then mapped onto each of the individual subjects of the current study (n = 64).

For the ROI analyses, average blood oxygenation level-dependent (BOLD) responses were extracted from each individual for the 8 ROIs (amygdala, hippocampus, anterior insula, dmPFC, thalamus, caudate, precuneus, and angular gyrus). For the primary analyses focused on the two MTL regions, the amygdala and hippocampus, repeated-measures ANOVAs were conducted for the left and right amygdala and hippocampus respectively, with condition (CS+ vs. CS−) as the within-group factor and group (Pers vs. NoPers) as the between-group factor. The ANOVAs were then repeated with depression, state anxiety scores, and APSS status added respectively as covariates. Similar analyses were conducted with the 6 additional, secondary ROIs.

### Correlations

To determine whether there was a dimensional relationship between the severity of psychotic experiences and MTL responses during fear conditioning across the full cohort, correlations between PDI total score and the fear conditioning responses in the bilateral hippocampus and amygdala were explored using bisquare robust regression models.

## Results

### Skin conductance responses during fear conditioning

As expected, the full cohort, as well as both the Pers and NoPers groups separately, exhibited physiological evidence for differential fear conditioning, i.e., significantly greater skin conductance responses (SCRs) to the CS+ compared to the CS− (all *p*s < 0.05; Supplementary Fig. 1), with no significant difference in fear conditioning magnitude between the Pers and NoPers groups (*t* = − 1.422, *p* = 0.177).

### Responses of the MTL during fear conditioning

**Figure 2:**
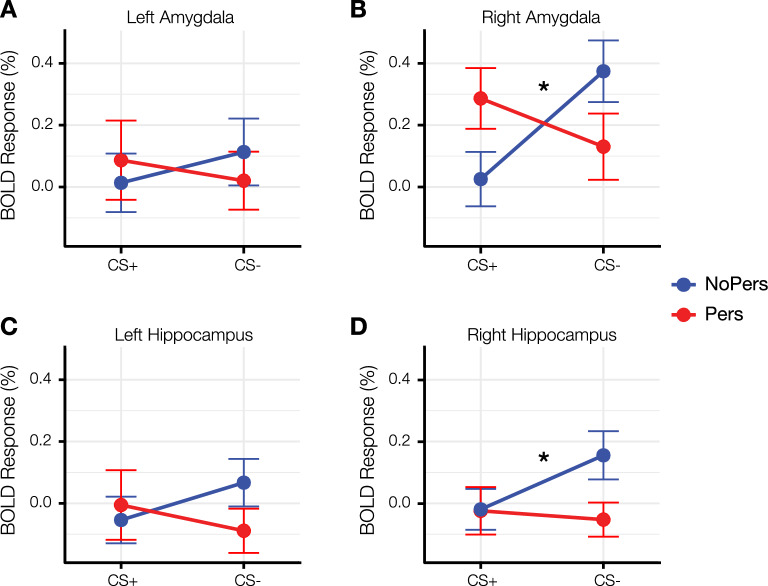
Amygdala and hippocampal responses of the Pers and NoPers groups during fear conditioning. The average responses of the amygdala and hippocampus to the CS+ and CS− of the Pers and NoPers groups, relative to a baseline condition (responses to a uniformly gray screen displaying a central fixation cross), are plotted. The Pers and NoPers groups exhibited distinct response patterns of the right amygdala and hippocampus during fear conditioning, as reflected by significant group by condition interactions (right amygdala (**B**): *p* = .001; right hippocampus (**D**): *p* = .012, indicated by an asterisk *). These effects were not observed in the left hemisphere (left amygdala (**A**); left hippocampus (**C**); *p*s > .05 after controlling for psychiatric symptomatology). Error bars represent one standard error above and below the mean.

A repeated-measures ANOVA revealed a significant interaction between condition and group for the right amygdala (*F*[1, 60] = 12.890, *p* < 0.001), left hippocampus (*F*[1, 60] = 4.258, *p* = 0.043) and right hippocampus (*F*[1, 60] = 4.251, *p* = 0.043) but not the left amygdala (*F*[1, 60] = 2.332, *p* = 0.132; Fig. 2). The interaction between condition and group for the right amygdala and right hippocampus remained significant (*p* < 0.05) after controlling for potential confounding factors, such as anxiety, depression and APSS status; whereas the group by condition interaction for the left hippocampus was no longer significant after controlling for these potential confounds (Table 2).

**Table 2:** Results of the analyses of variance controlling for potential confounds.

Region	Covariate	Left hemisphere			*R*^*2*^	Right hemisphere			*R*^*2*^
		*F*	*df*	*p*		*F*	*df*	*p*	
Amygdala	–	2.33	1.62	0.132	0.036	12.89	1.62	** < 0.001**	0.172
	Depressive symptoms	1.19	1.61	0.280	0.047	15.53	1.61	** < 0.001**	0.203
	State anxiety	2.31	1.61	0.133	0.037	14.65	1.61	** < 0.001**	0.196
	Attenuated positive symptom syndrome	1.29	1.61	0.260	0.056	11.53	1.61	**0.001**	0.172
Hippocampus	–	4.26	1.62	**0.043**	0.064	4.25	1.62	**0.043**	0.064
	Depressive symptoms	2.63	1.61	0.110	0.072	5.21	1.61	**0.026**	0.079
	State anxiety	3.68	1.61	*0.060*	0.066	6.08	1.61	**0.017**	0.111
	Attenuated positive symptom syndrome	3.61	1.61	*0.062*	0.065	3.88	1.61	*0.053*	0.064

Depression was measured with the Beck Depression Inventory; state anxiety was measured with the State-Trait Anxiety Inventory; and the presence of the Attenuated Positive Symptom Syndrome (APSS) was measured with the Structured Interview for Psychosis-Risk Syndromes. P values for group by condition interaction for a region (with the included covariate listed on the left) are listed, with the significant values (*p* < 0.05) bolded and the trend level p values (*p* < 0.1) italicized. The R^2^ values listed represent the proportion of variance of the dependent variable that can be explained by the independent variable. After controlling for these potential confounding variables, the findings for the right amygdala and hippocampus remained significant or near significant (*p* = 0.053 for the right hippocampus when controlling for APSS status).

Follow-up t-tests revealed that the NoPers group (Fig. 2) exhibited the expected pattern of responses in the right amygdala (*t* = 4.416, *p* < 0.001, *d* = 0.535) and right hippocampus (*t* = 3.672, *p* = 0.001, *d* = 0.349), with significantly larger responses to the CS− compared to the CS+. In contrast, the Pers group failed to show this response in either the right amygdala or right hippocampus (Fig. 2; *p*s > 0.05). A between-group comparison of the responses of these two regions further confirmed this finding, showing a significantly greater CS− vs. CS+ response of the right amygdala (*t* = 3.566, *p* = 0.001, *d* = 0.328) and right hippocampus (*t* = 2.595, *p* = 0.012, *d* = 0.370) in the NoPers compared to the Pers group.

### Secondary analyses

The analysis of the six additional ROIs revealed that the Pers group and the NoPers group (Supplementary Figs. 2 and 3) each exhibited differential fear conditioning-related responses in the bilateral anterior insula, dmPFC, caudate nucleus, and thalamus (with CS+  > CS− responses), as well as in the precuneus and angular gyrus (with CS− > CS+ responses) (all *p*s < 0.05; see Supplementary Table 1). Direct comparisons between the Pers and NoPers groups in the magnitude of fear conditioning-related responses in these regions showed that there were significant between-group differences in the left caudate nucleus and bilateral default mode network regions (i.e., angular gyrus and precuneus; *p*s < 0.05; see Supplementary Table 1), but not in the anterior insula, thalamus or dmPFC (all *p*s > 0.05; see Supplementary Table 1).

### Symptom correlations

In the full cohort (n = 64), there was a significant negative correlation between the severity of psychotic experiences (total PDI score) and the magnitude of differential conditioning (CS− > CS+) responses of the bilateral amygdala (*b* = −0.026, *t* = 2.550, *p* = 0.013) and bilateral hippocampus (*b* = −0.013, *t* = 2.180, *p* = 0.033). There were no such correlations in the Pers and NoPers groups alone, with the separate responses of the right or left amygdala or hippocampus, or between levels of anxiety or depression and MTL fear conditioning-related responses in the full cohort or either of the two groups (all *p*s > 0.05).

## Discussion

### Summary of main findings

In this study, non-help-seeking young adults who endorsed persecutory beliefs showed abnormal neural responses during Pavlovian fear conditioning when compared to demographically matched adults without such beliefs. Specifically, the expected pattern (CS− > CS+) of learned responses^36,37^ was observed in the participants without persecutory beliefs in the right amygdala and hippocampus, but not in those with persecutory beliefs. In addition, the severity of psychotic experiences across the full sample correlated with the magnitude of this abnormality.

### Fear conditioning responses in the human medial temporal lobe

We found that the participants without persecutory beliefs (the control group) showed greater responses to the CS− than the CS+ in the amygdala and hippocampus, which is consistent with the results of some but not all prior studies. In particular, prior fMRI studies of fear conditioning conducted in healthy humans have shown mixed findings with respect to the role of the amygdala, with early studies showing greater response to the CS+ than the CS−^34,35^, but later studies detecting only transient or inconsistent amygdala responses during fear conditioning^36^. A meta-analysis of fMRI studies of fear conditioning in humans (including 27 studies, N = 677) did not detect significant amygdala responses during fear conditioning but identified robust CS− > CS+ responses within the hippocampus and closely connected areas such as the posterior cingulate cortex^36^. A second meta-analysis found similar results, observing strong CS− > CS+ responses in both the amygdala and hippocampus during fear conditioning^37^. Moreover, another recent study aiming to resolve these questions about the role of the amygdala in human fear conditioning (N = 601) suggested that the responses of the amygdala during fear conditioning likely depends on temporal factors and varies across subnuclei of the amygdala, with late (versus early) conditioning in the basolateral nucleus of the amygdala favoring “safety” (CS− > CS+) over “threat” (CS+  > CS−) signaling^70^. Thus, the authors of this study concluded that, due to the functional heterogeneity of the amygdala, robust CS+  > CS− amygdala responses may only be detectable with large sample sizes^70^. Despite the variability of prior findings, the overall literature suggests that the amygdala and hippocampus are involved in both types of responses, i.e., threat and safety -related signaling^39,40^. In a non-threatening context, safety signaling may dominate^39^, and safety-selective neurons of the amygdala and hippocampus may respond preferentially to the CS− in Pavlovian fear conditioning paradigms^38,39,41^. Prior studies have also shown that default network regions show greater responses to the CS− compared to the CS+ ^71^, consistent with overall evidence that there is suppression of default network activity in the presence of behaviorally-salient stimuli in the environment^72,73^, including those associated with an electric shock (a CS+).

### Impaired safety signaling in psychosis

Thus, taken together, one interpretation of the current results is that individuals with (or at risk for developing) persecutory beliefs are impaired in their capacity to generate one type of safety signal, which may render them vulnerable to worsening psychotic symptoms, particularly in certain environmental contexts associated with elevated levels of stress. This finding is generally consistent with prior evidence that another form of associative memory-based safety signaling, the recall of extinction memory traces, is impaired in individuals diagnosed with schizophrenia^42^, particularly those with delusions^30^. Thus, the function of a network of brain regions involved in both the encoding and retrieval of safety-related information may be altered in individuals who are vulnerable to or experiencing psychotic symptoms, such as persecutory delusions.

### Abnormal responses of the default network

In addition to a reduction in the CS− > CS+ responses of the amygdala and hippocampus, the participants with persecutory beliefs also demonstrated a similar pattern of impaired CS− > CS+ responses of two default network areas, the precuneus and angular gyrus, compared to the control group. These findings are consistent with those of a prior fear conditioning fMRI study of a combined sample of individuals with attenuated psychotic symptoms and clinical psychosis, which detected lower CS− > CS+ responses of another default network region, the ventromedial prefrontal cortex (a region showing significant CS− > CS+ responses in the healthy control subjects), in the psychosis spectrum group compared to the control group^74^. This pattern of poor engagement of default network areas during the less behaviorally salient condition (the CS− in this case) may be related to observations of impaired task-induced suppression of default network areas reported previously in studies using cognitively demanding tasks (e.g., working memory paradigms) in schizophrenia^72,73^ and psychosis risk^75^. Thus, an alternative interpretation of the current findings is that attentional impairment, or some other cognitive deficit, may have led to reduced engagement of default network and medial temporal lobe areas during the non-threatening condition (the CS−). Although there were no differences between the groups in accuracy in the low-level attentional task that was performed during the fear conditioning paradigm (detecting brief head nods of the face stimuli), this task may not have been engaging enough to consistently capture the attention of participants throughout data collection.

Alternatively, given that the hippocampus and precuneus are well-established components of an extended episodic memory network, the CS− > CS+ response may reflect the encoding of an episodic memory trace related to contingency awareness (the CS—shock or no shock association), and this memory encoding may be disrupted in individuals with persecutory beliefs^30,36^. Future studies which measure default network and medial temporal lobe responses using both fear conditioning and other types of tasks can determine whether the changes observed in the current study represent a specific safety signaling deficit related to impaired associative learning or another type of cognitive impairment, a broader abnormality in default network and medial temporal lobe functioning, or both.

### CS+  > CS− responses

In contrast to the pattern observed in the amygdala, hippocampus, and default network, the caudate nucleus showed significantly greater responses to the CS+ compared to the CS−, and the subjects with persecutory beliefs exhibited blunting of this response in the left caudate nucleus. This pattern is consistent with a finding of reduced differential conditioning (lower CS+  > CS−) in the physiological expression (skin conductance responses) of fear conditioning in a recent meta-analysis that compared 77 schizophrenia patients to 74 controls^29^. Given that a deficient skin conductance response during fear conditioning was not evident in the subjects with persecutory beliefs in the current study, we speculate that an abnormality in this peripheral measure of fear conditioning may only arise at a later stage of illness, or may be present in only those who will later develop psychotic illness.

However, this pattern of results is reminiscent of those of a number of prior studies of associative learning in psychotic disorders that found abnormalities in neural responses accompanied by intact behavioral responses indicating successful associative learning in the psychosis group^24,76^. Murray and colleagues found evidence for several possible explanations for this dissociation^24^, including that low-level engagement of the relevant neural circuitry, or compensatory recruitment of areas outside of the hypothesized brain network, may have been sufficient for normal task performance in these prior studies. Also, fMRI may be more sensitive to subtle differences in associative learning mechanisms than behavioral outcomes. In the current study, the absence of differences between the two groups in the responses during fear conditioning of the majority of the salience network regions, including the insula, dorsomedial prefrontal cortex and thalamus, is consistent with these explanations; autonomic responses during fear conditioning may be generated primarily by this network^26^, and may be relatively intact in non-help-seeking young people with persecutory beliefs.

The reduction in striatal responses in the group with persecutory beliefs during fear conditioning is consistent with a number of prior fMRI studies showing reduced striatal responsiveness in individuals with attenuated psychotic symptoms across a number of experimental paradigms^77–79^, suggesting that this finding may be task-independent, or that a fundamental associative learning deficit underlies the striatal dysfunction observed across these studies.

### Relationship of these findings to animal models of psychosis

The findings of this study are in line with a well-known model of psychosis, based on pre-clinical studies conducted primarily in rodents, which posits that overactivity of the hippocampus, due to impaired functioning of hippocampal inhibitory interneurons, leads to disruption of the function of the striatum and closely connected regions of the basal ganglia and midbrain^80^. Findings of several imaging studies conducted in individuals with schizophrenia^80^ or with attenuated psychosis^6^ have provided support for this model. For example, overactivity of the hippocampus in individuals who are at risk for psychosis (who have attenuated psychotic symptoms) has been linked to overactivity of regions involved in dopamine signaling, including the midbrain and basal ganglia^6,7,9^. The current results suggest that psychotic symptoms, particularly persecutory beliefs, may arise from impairments in associative learning that are linked to dysfunction of this hippocampal-striatal circuitry.

### Limitations

There are several limitations of this work that must be considered when interpreting these findings. First, the participants of this study did not undergo diagnostic evaluations for psychiatric disorders; thus we cannot exclude the possibility that individuals with diagnoses of psychotic disorders or other psychiatric illnesses were included in this sample. However, the fact that no participants received a score of 6 on any of the positive symptom items of the assessment of psychosis risk (indicating the presence of active clinical psychosis) confirms that no actively psychotic participants were included. Also, no participants were being treated with psychotropic medications, and all were enrolled in college as full-time students at the time of the study, suggesting that, if psychopathology was present, it was not severe or disabling.

In addition, in future studies, the inclusion of a clinical comparison group, i.e., those with schizophrenia or a related psychotic illness, would allow for a comparison of fear conditioning responses of those with mild persecutory beliefs with those with more severe and impairing persecutory beliefs that are held with conviction. We elected not to include such a group in this initial study because of the potentially confounding effects of treatment with antipsychotic medication and the effects on the brain of an ongoing disabling illness that can be present in a clinical psychosis sample. Future studies in larger samples can investigate the degree of overlap between medial temporal lobe-based associative memory abnormalities found in those with subclinical versus clinical levels of psychotic symptoms.

### Additional future directions

In this study, deficient responses of regions of a medial temporal lobe-default-striatal network were observed during fear conditioning in a non-help-seeking sample of young adults who endorsed persecutory beliefs, when compared to a well-matched group of young adults without such beliefs. These findings could provide further justification for specific mechanistic studies of psychosis. For example, studies of a GAD65 gene knock-out mouse, which lacks the capacity for GAD65-mediated GABA synthesis, have shown that this alteration leads to abnormal fear conditioning-related responses^81,82^ and hyperactivity of the amygdala, hippocampus, and medial hypothalamus^83^. These findings suggest that deficits in GABAergic functioning could potentially account for the abnormalities observed in the current study. Future studies could directly test this model, by measuring levels of GABA and fear conditioning responses of the medial temporal lobe-default-striatal circuit in individuals with and without persecutory beliefs.

In addition, the specific pattern of disrupted fear and safety learning observed in this study could be tested as a potential marker of risk for clinical psychosis in longitudinal studies that measure the development of psychotic disorders in those at elevated risk for such disorders. A subset or combination of risk factors (e.g., attenuated psychotic symptoms/persecutory beliefs, polygenic risk score for psychosis or transdiagnostic conditions, environmental stressors) may be linked to such deficits in associative memory processes. The identification of a neural correlate of a psychotic symptom in an unmedicated, treatment-free sample, consistent with a neural systems model of psychosis, represents a rational candidate for further testing as a potential biomarker of psychosis risk.

### Supplementary Information

Supplementary Information.

## Data Availability

The datasets used in the analyses of this study are available from the corresponding author by request.
