# Author Correction: Changes in responses of the amygdala and hippocampus during fear conditioning are associated with persecutory beliefs

**DOI:** 10.1038/s41598-024-60109-3

**Published:** 2024-04-23

**Authors:** Wisteria Deng, Lauri Tuominen, Rachel Sussman, Logan Leathem, Louis N. Vinke, Daphne J. Holt

**Affiliations:** 1 Department of Psychiatry, Massachusetts General Hospital, 149 13Th, St. Charlestown, Boston, MA 02129 USA; 2 Department of Psychology, Yale University, New Haven, CT USA; 3 Department of Psychiatry, University of Ottawa, Ottawa, ON Canada; 4 Department of Psychiatry, Harvard Medical School, Boston, MA USA; 5 Athinoula A. Martinos Center for Biomedical Imaging, Massachusetts General Hospital, Boston, MA USA

Correction to: *Scientific Reports* 10.1038/s41598-024-57746-z, published online 08 April 2024

The original version of this Article contained errors. In the Introduction,

“Subclinical psychotic symptoms, often referred to as “psychotic experiences,” are common in the general population and are typically non-distressing and transient^35^. Although the majority of individuals with psychotic experiences do not go on to develop frank psychosis, there is converging evidence, based on studies of epidemiologic and environmental risk factors for clinical psychosis^42,43^, as well as neuroimaging data^44,45,46,47^, for some degree of a continuum in the clinical and neurobiological expression of psychosis across different levels of severity in the general population, and for some biological mechanisms that are shared across psychotic experiences and clinical psychosis.”

now reads:

“Subclinical psychotic symptoms, often referred to as “psychotic experiences,” are common in the general population and are typically non-distressing and transient. Although the majority of individuals with psychotic experiences do not go on to develop frank psychosis, there is converging evidence, based on studies of epidemiologic and environmental risk factors for clinical psychosis^43^, as well as neuroimaging data^44,45,46,47^, for some degree of a continuum in the clinical and neurobiological expression of psychosis across different levels of severity in the general population, and for some biological mechanisms that are shared across psychotic experiences and clinical psychosis.”

In the Methods, under the subheading ‘Recruitment of participants’,

“Consistent with this, in our prior studies using these screening methods, these criteria typically identified approximately 20% (with elevated PDI scores) and 48% (with elevated BDI scores) of the distribution of the college students screened^50,58^.”

“To test our specific hypothesis about persecutory beliefs, the enrolled cohort was divided into those who endorsed persecutory beliefs (one or both of the two persecutory items of the PDI)^51,53^ and those who did not.”

now reads:

“Consistent with this, in our prior studies using these screening methods, these criteria typically identified approximately 20% (with elevated PDI scores) and 48% (with elevated BDI scores) of the distribution of the college students screened^49,50^.”

“To test our specific hypothesis about persecutory beliefs, the enrolled cohort was divided into those who endorsed persecutory beliefs (one or both of the two persecutory items of the PDI) and those who did not.”

Under subheading ‘Clinical measures’,

“We measured persecutory beliefs using the previously identified “persecutory factor” of the PDI^64^, which includes two items: “Do you ever feel as if you are being persecuted in some way?” (item #4) and “Do you ever feel as if there is a conspiracy against you?” (item #5).”

now reads:

“We measured persecutory beliefs using the previously identified “persecutory factor” of the PDI^55,64,66^, which includes two items: “Do you ever feel as if you are being persecuted in some way?” (item #4) and “Do you ever feel as if there is a conspiracy against you?” (item #5).”

In the Discussion, under the subheading ‘Summary of main findings’,

“Specifically, the expected pattern (CS− > CS+) of learned responses^34,35^ was observed in the participants without persecutory beliefs in the right amygdala and hippocampus, but not in those with persecutory beliefs. In addition, the severity of psychotic experiences across the full sample correlated with the magnitude of this abnormality.”

now reads:

“Specifically, the expected pattern (CS− > CS+) of learned responses^36,37^ was observed in the participants without persecutory beliefs in the right amygdala and hippocampus, but not in those with persecutory beliefs. In addition, the severity of psychotic experiences across the full sample correlated with the magnitude of this abnormality.”

Under the subheading ‘Fear conditioning responses in the human medial temporal lobe’,

“Despite the variability of prior findings, the overall literature suggests that the amygdala and hippocampus are involved in both types of responses, i.e., threat and safety -related signaling^39,40,69^.”

now reads:

“Despite the variability of prior findings, the overall literature suggests that the amygdala and hippocampus are involved in both types of responses, i.e., threat and safety -related signaling^39,40^.”

Under the subheading, ‘Relationship of these findings to animal models of psychosis’,

“Findings of several imaging studies conducted in individuals with schizophrenia^81^ or with attenuated psychosis^6^ have provided support for this model.”

now reads:

“Findings of several imaging studies conducted in individuals with schizophrenia^80^ or with attenuated psychosis^6^ have provided support for this model.”

Under the subheading, ‘Additional future directions’,

“For example, studies of a GAD65 gene knock-out mouse, which lacks the capacity for GAD65-mediated GABA synthesis, have shown that this alteration leads to abnormal fear conditioning-related responses^82^ and hyperactivity of the amygdala, hippocampus, and medial hypothalamus^83^.”

now reads:

“For example, studies of a GAD65 gene knock-out mouse, which lacks the capacity for GAD65-mediated GABA synthesis, have shown that this alteration leads to abnormal fear conditioning-related responses^81,82^ and hyperactivity of the amygdala, hippocampus, and medial hypothalamus^83^.”

The original Article has been corrected.

