# Supplementary material for: Changes in responses of the amygdala and hippocampus during fear conditioning are associated with persecutory beliefs
Source: Sci Rep. 2024 Apr 8;14:8173. doi: 10.1038/s41598-024-57746-z (PMC11001942; doi:10.1038/s41598-024-57746-z)
Supplement: Supplementary file 1 — Supplementary Information. [file 41598_2024_57746_MOESM1_ESM.docx]

|  | | |  | |  | |  | |  |  |  |  |  |
| --- | --- | --- | --- | --- | --- | --- | --- | --- | --- | --- | --- | --- | --- |
|  |  |  | | Left hemisphere Right hemisphere | | | | | | | | | |
| Region | | | *df* | | | *F* | | *p* | | *df* | *F* | *p* |  |
| Insula | | | 1,62 | | | .190 | | .664 | | 1,62 | .001 | .976 |  |
| dmPFC | | | 1,62 | | | .141 | | .708 | | 1,62 | 1.260 | .266 |  |
| Thalamus | | | 1,62 | | | 2.164 | | .146 | | 1,62 | .609 | .438 |  |
| Caudate | | | 1,62 | | | 4.266 | | **.043** | | 1,62 | 2.827 | *.098* |  |
| Precuneus | | | 1,62 | | | 7.358 | | **.009** | | 1,62 | 4.738 | **.033** |  |
| Angular gyrus | | | 1,62 | | | 6.674 | | **.012** | | 1,62 | 6.705 | **.012** |  |

**Supplementary Table 1.** Results of the analyses of variance for the Salience Network and Default Network regions (the secondary analysis). P values for group (Pers, NoPers) by condition (CS+, CS-) interactions are listed in the righthand column. Significant p values (p < .05) are bolded and trend-level p values (p < .1) are italicized. Direct comparisons between the Pers and NoPers groups in the magnitude of fear conditioning-related responses in these regions showed that there were significant between-group differences in the left caudate nucleus and bilateral default mode network regions (angular gyrus and precuneus), but not in the anterior insula, thalamus or dorsomedial prefrontal cortex (dmPFC). Pers = group with persecutory beliefs (*n* = 21); NoPers = group without persecutory beliefs (*n* = 43).


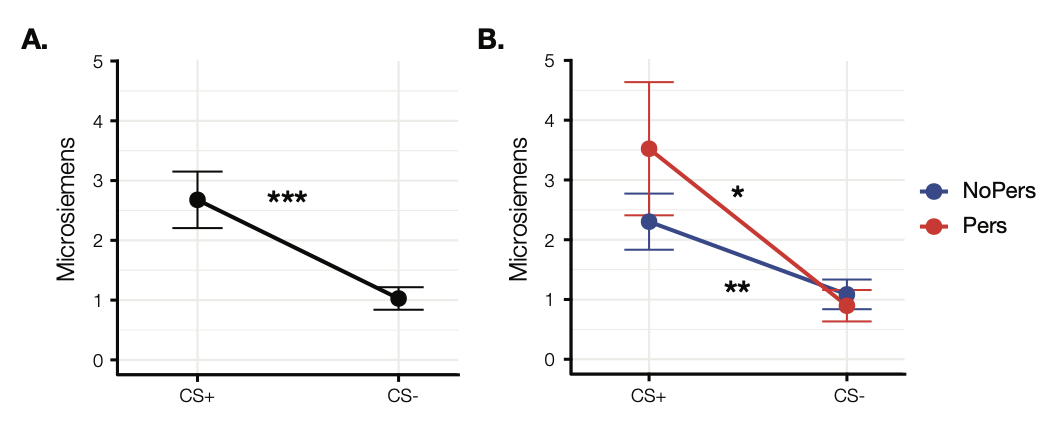


**Supplementary Figure 1.** Mean skin conductance responses (SCRs) during fear conditioning. The SCR data reveal that the full cohort (A, *n* = 39) and both groups (B, *n* = 12 and 27 for the Pers and NoPers groups, respectively) showed significant, differential conditioned fear responses. Error bars represent standard errors of the mean. P-values of paired t-tests: *p < .05, **p < .01, ***p < .001.


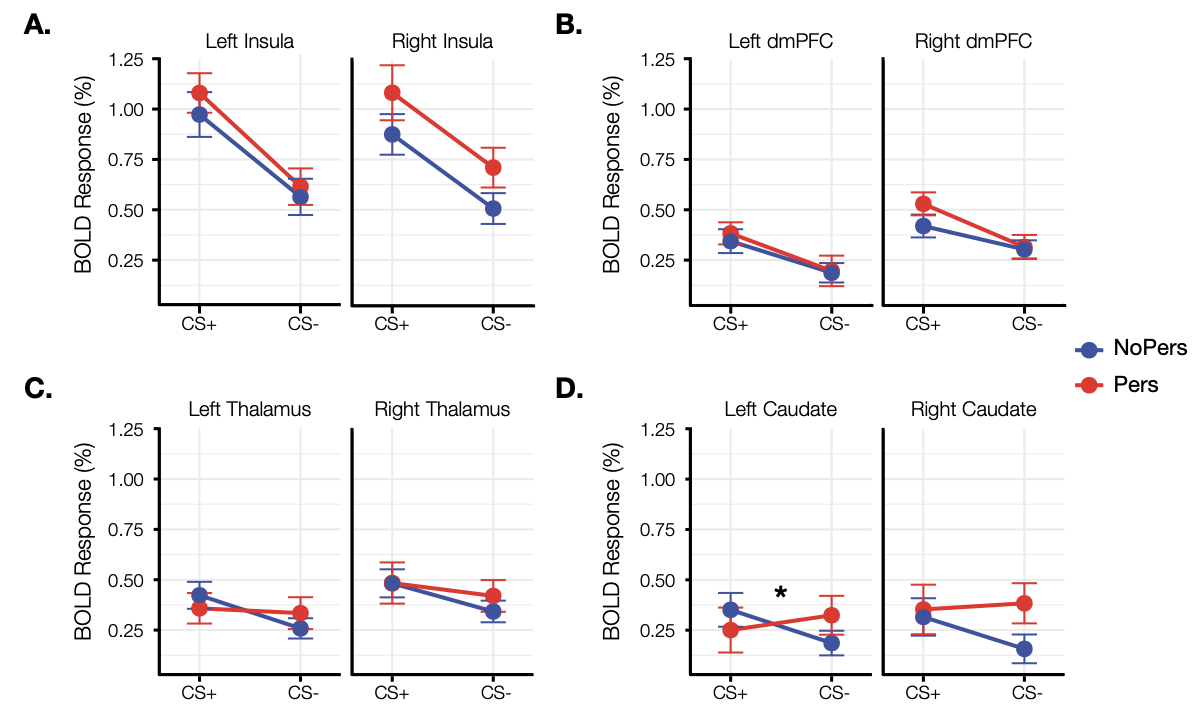


**Supplementary Figure 2.** Fear conditioning-related Blood Oxygenation Level-Dependent (BOLD) responses of the Salience Network. The average BOLD responses to the CS+ and CS- of the Pers and NoPers groups, relative to a baseline condition (responses to a uniformly gray screen displaying a central fixation cross), are plotted. In the full cohort (N = 64, data not shown here), differential fear conditioning-related responses (all p >.05) were observed in multiple regions of the Salience Network (anterior insula, dorsomedial prefrontal cortex (dmPFC), thalamus and caudate nucleus). When the responses of these regions within the separate Pers and NoPers groups were directly compared, there were no significant differences between the two groups in fear-conditioning-related BOLD responses, except in the left caudate nucleus. *p < .05 for independent samples t-test. Pers = group with persecutory beliefs (*n* = 21); NoPers = group without persecutory beliefs (*n* = 43). The y-axis represents the average BOLD responses as percent signal change. Error bars represent standard errors of the mean.


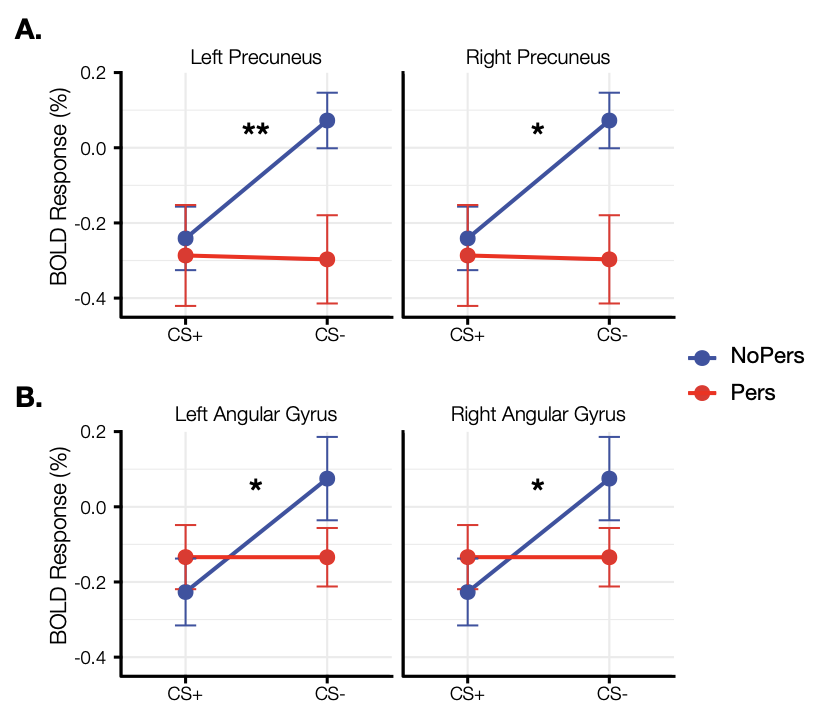


**Supplementary Figure 3.** Fear conditioning-related Blood Oxygenation Level-Dependent (BOLD) responses of the Default Network. The average BOLD responses to the CS+ and CS- of the Pers and NoPers groups, relative to a baseline condition (responses to a uniformly gray screen displaying a central fixation cross), are plotted. In the full cohort (N = 64, data not shown here), differential fear conditioning-related BOLD responses (all p >.05) were observed in two regions of the default network, the precuneus and angular gyrus. When the responses of these regions of the Pers and NoPers groups were directly compared using independent samples t-tests, there were significant differences between the two groups in fear-conditioning-related BOLD responses of these two regions. P-values of independent samples t-tests: *p < .05, **p < .01. Pers = group with persecutory beliefs (*n* = 21); NoPers = group without persecutory beliefs (*n* = 43). The y-axis represents the average BOLD responses as percent signal change. Error bars represent standard errors of the mean.
